# Ipsilateral femoral neck and shaft fracture in children: a report of two cases and a literature review

**DOI:** 10.1007/s10195-012-0188-9

**Published:** 2012-05-05

**Authors:** Kwang Soon Song, Kirti Ramnani, Chul Hyun Cho, Ki Cheor Bae, Kyung Jae Lee, Eun Seok Son

**Affiliations:** Department of Orthopedic Surgery, Keimyung University, 194 Dondsandong, Joong-gu, Daegu, 700-712 Korea

**Keywords:** Femoral neck fracture, Ipsilateral femoral shaft fracture, Children

## Abstract

Concomitant ipsilateral fractures of the neck and shaft of the femur in children are rare. The most recent report in this context found a total of only nine reported cases (<12 years of age) following a search of the indexed English literature. These injuries occur in children due to high-velocity trauma, and there is no generally accepted method of treatment. We report three additional cases from the literature and two cases of our own. In our cases, one had a residual 10° varus deformity at the subtrochanteric level in the femur, but this did not affect hip function. Another patient exhibited a limp at final follow-up due to leg length discrepancy, and peroneal nerve palsy at the time of injury. We advocate operative stabilization of the femoral shaft fracture first to reduce the risk of further displacement and simplify the subsequent reduction of the femoral neck. The series shows that these rare injuries have a poor prognosis, with high rates of incidence of avascular necrosis, coxa vara, and leg length discrepancy.

## Introduction

Concomitant ipsilateral fractures of the neck and shaft of the femur in children are rare. The authors of the most recent report in this context found that only nine cases (<12 years of age) have been reported to date, including two of their own cases; the other seven cases were found by searching the indexed English literature [1]. Only one or two cases are reported in each of the works they found in the literature [1–7]. These injuries occur in children due to high-velocity trauma, and there is no universally accepted method of treatment [8]. We report two additional cases with such a combination of injuries as well as three additional reports [9–11] found in the literature. Our study showed that there is a relatively high complication rate with transepihyseal or transcervical femoral neck fractures, so we disagree with the opinion that a symbiotic effect during the time of injury results in a lower incidence of complications [1]. The present study was performed after obtaining informed consent from the patients’ parents or guardians.

## Case reports

### Case 1

**Fig. 1:**
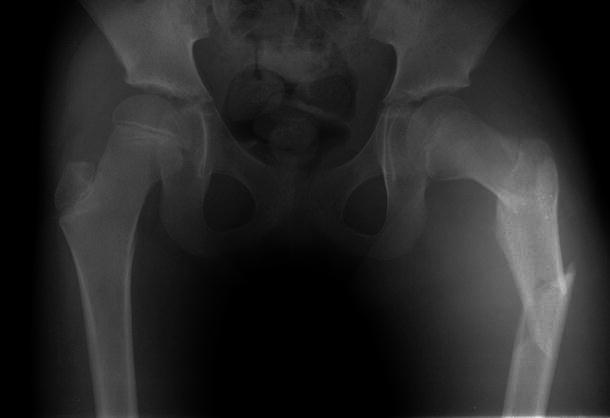
Eight-year-old girl: anteroposterior radiographs reveal a cervicotrochanteric fracture (type III in Delbet’s classification; AO classification type II) with a subtrochanteric fracture of the left femur

**Fig. 2:**
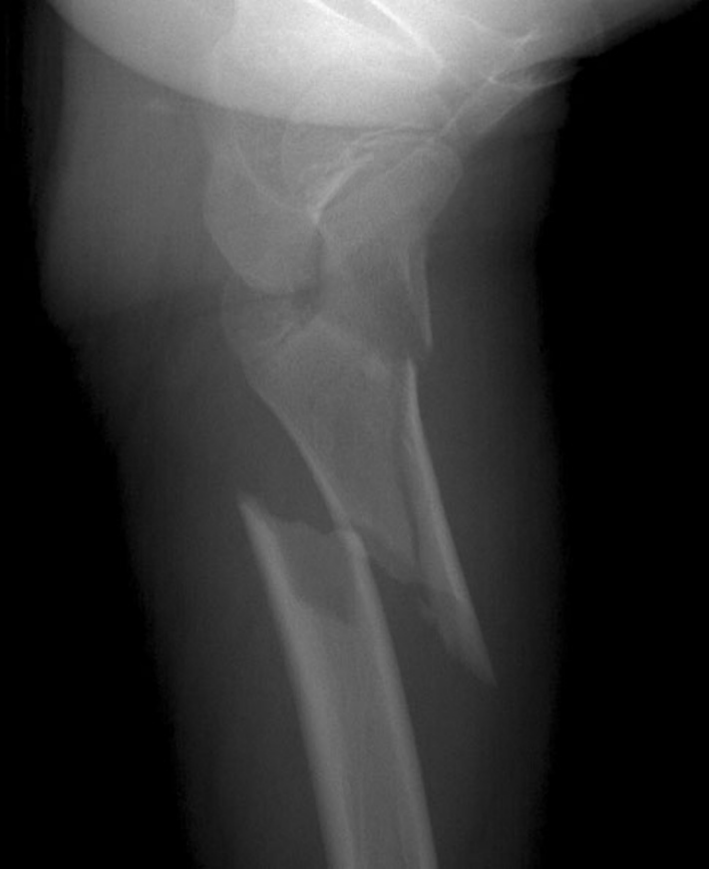
Eight-year-old girl: lateral radiographs reveal a cervicotrochanteric fracture (type III in Delbet’s classification; AO classification type II) with a subtrochanteric fracture of the left femur

**Fig. 3:**
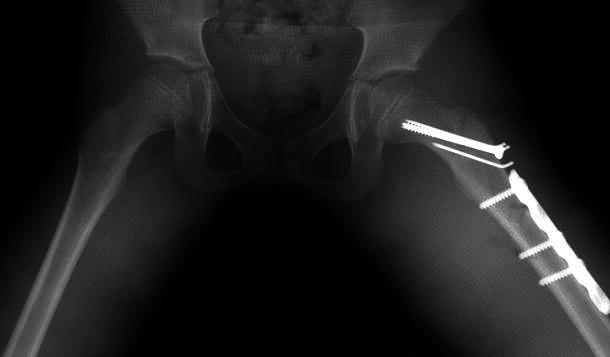
The neck femur fracture was fixed with a single cannulated screw 3.5 mm in diameter and three Steinmann pins 2 mm in diameter, the subtrochanteric fracture was fixed with a five-hole compression plate, and the proximal fragment was fixed with a single screw to avoid impingement during fixation of the femoral neck fracture

**Fig. 4:**
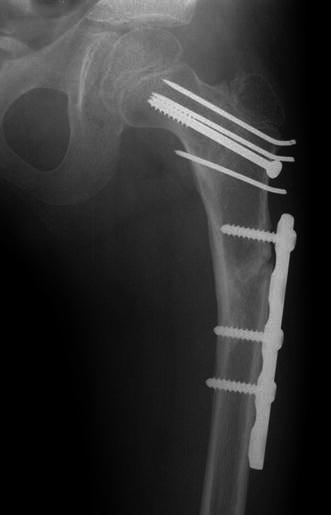
After removing the cast at two months postoperatively, the radiograph showed a 10° varus deformity of the subtrochanteric fracture with proximal screw back-out

**Fig. 5:**
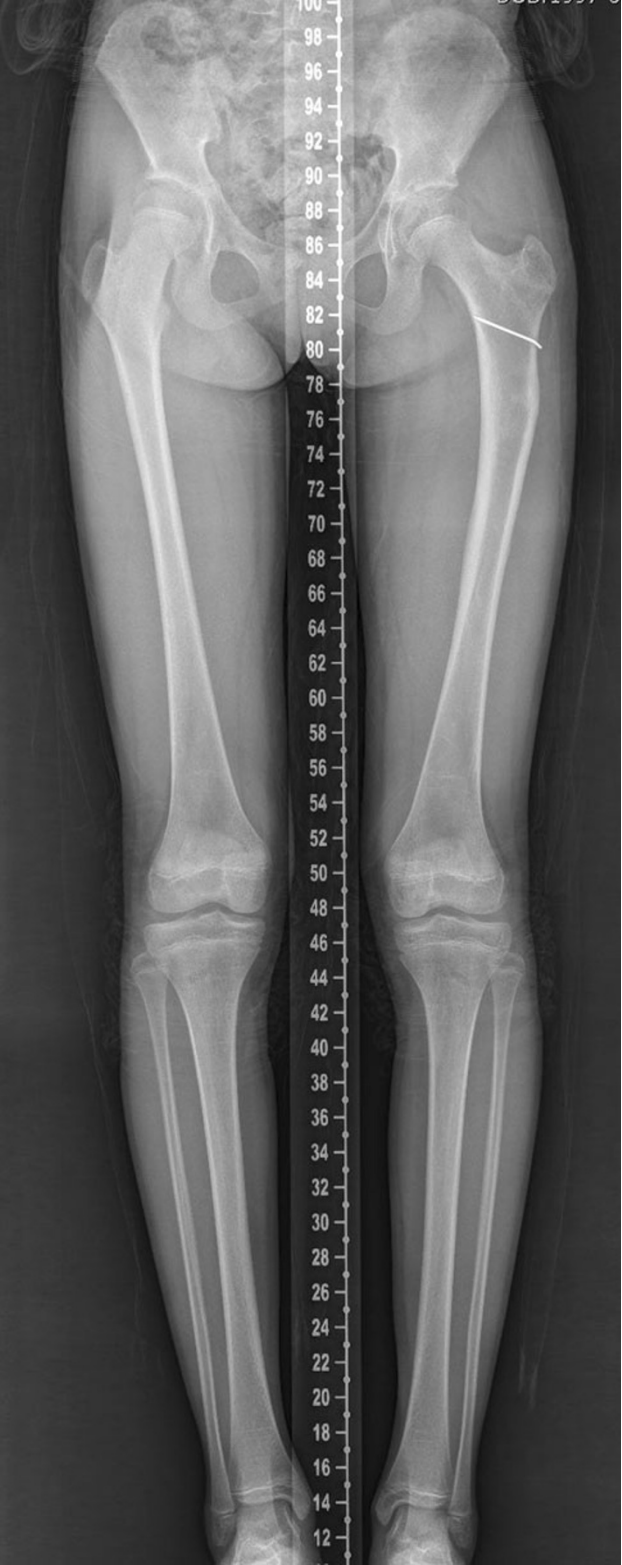
Telereontgenogram shows no evidence of avascular necrosis of the femoral head or growth disturbance except for the residual 10° varus deformity at the subtrochanteric level in the femur

An eight-year old girl was run over by a car and was subsequently admitted to the hospital with an obvious deformity and swelling in the left thigh. Radiograph revealed a cervicotrochanteric fracture (type III in Delbet’s classification [12]; AO classification type II [13]) with a subtrochanteric fracture of the left femur (Figs. 1, 2). Surgery was performed 10 h after the trauma, after obtaining medical clearance for anesthesia. The treatment included open reduction and plate fixation of the subtrochanteric fracture, and closed reduction and internal fixation of the femoral neck with a screw and Steinmann pins. The proximal fragment of the subtrochanteric fracture was held firmly with bone-holding forceps to prevent further displacement of the femoral neck fracture during reduction and application of the plate. Although the subtrochanteric fracture was fixed with a five-hole compression plate, the proximal fragment was fixed with a single screw to avoid impingement during fixation of the femoral neck fracture. The bone-holding forceps were retained until closed reduction and fixation of the femoral neck fracture had been achieved with a single cannulated screw 3.5 mm in diameter and three Steinmann pins 2 mm in diameter (Fig. 3). Postoperatively, the child was immobilized in a one-and-a-half hip spica cast (hip flexion 30° and abduction 40° for both hips, and knee flexion 30° for the affected limb) to augment the stability of the subtrochanteric fracture fixation. However, after removal of the cast at two months postoperatively, the radiograph showed a 10° varus deformity of the subtrochanteric fracture with proximal screw back-out (Fig. 4). We started non-weight-bearing mobilization exercises at two months and three weeks postoperatively. Walking exercises were started with an ischial weight-bearing long leg brace four months postoperatively, progressing to full weight bearing by five months. Implant removal was performed 11 months postoperatively. Follow-up at five years after the injury showed normal and painless hip function clinically; radiographs showed no evidence of avascular necrosis of the femoral head or growth disturbance, except for the residual 10° varus deformity at the subtrochanteric level in the femur (Fig. 5).

### Case 2

**Fig. 6:**
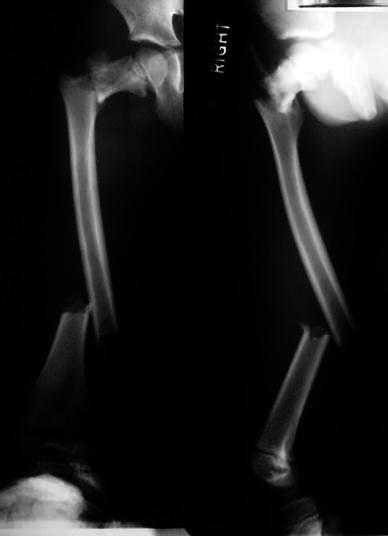
Boy seven years and ten months of age: anteroposterior and lateral radiographs reveal a right intertochanteric fracture (type IV in Delbet’s classification; AO classification type III) and an ipsilateral lower one-third fracture of the femoral shaft

**Fig. 7:**
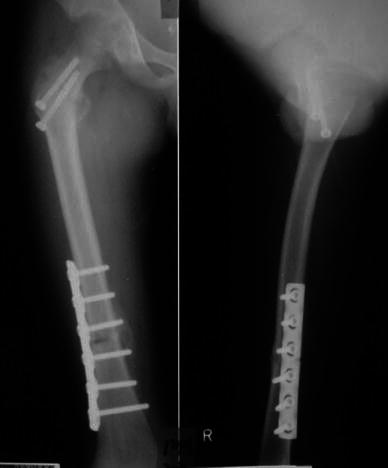
At six weeks after the operation, the radiographs show well-maintained reduction. The patient was started on non-weight-bearing active exercises

**Fig. 8:**
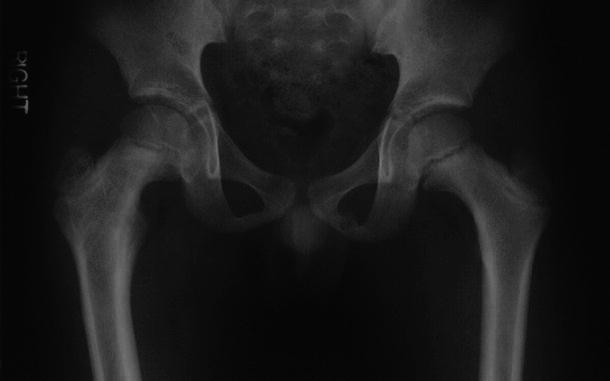
At two-year follow up, radiographs show union of the femoral neck fracture without avascular necrosis of the femoral head

**Fig. 9:**
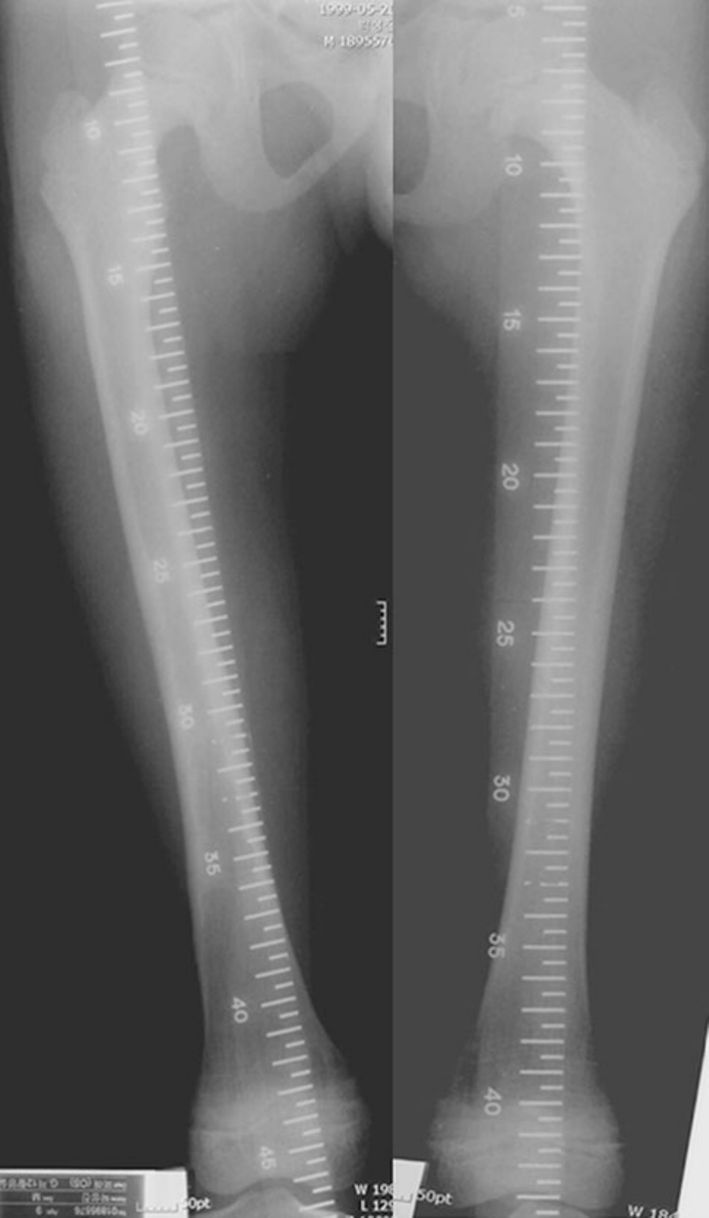
At two-year follow up, radiographs show overgrowth of the right femur of 1.5 cm

A boy seven years and ten months of age was involved in a head-on collision with car and admitted to the hospital in a drowsy state. Radiographs revealed a right intertochanteric fracture (type IV in Delbet’s classification; AO classification type III) and an ipsilateral lower one-third fracture of the femoral shaft (Fig. 6). He had additional injuries, including subdural brain hemorrhage, lateral condylar fracture of the right humerus, and left distal radius fracture. Immediate skeletal traction was applied to the ipsilateral tibia to prevent further displacement of the fracture and damage to the blood supply of the femoral head. Once the general condition of the patient had improved after 3 days, open reduction and internal fixation of the distal femur fracture with a six-hole dynamic compression plate and closed reduction and internal fixation of the femoral neck fracture with two cannulated screws 3.5 mm in diameter were performed. The proximal fragment was stabilized with bone-holding forceps during fixation of the femoral shaft fracture to avoid the inadvertent transmission of force to the neck fracture. Postoperatively, the patient was immobilized in a one-and-a-half hip spica cast (hip flexion 30° and abduction 30° for both hips, and knee flexion of 30° in the affected knee). We removed the hip spica cast and started non-weight-bearing active mobilization exercises at six weeks postoperatively (Fig. 7). Walking exercises were started with an ischial weight-bearing long leg brace three months postoperatively, progressing to full weight bearing by four months. Implant removal was done 1 year postoperatively. At the two-year follow-up, radiographs revealed that there was no avascular necrosis, but an overgrowth of 1.5 cm was noted (Figs. 8, 9). The patient walked with a limping gait as a sequela of the subdural brain hemorrhage, leg length discrepancy, and unrecovered peroneal nerve palsy.

## Discussion

Displaced femoral neck fractures are uncommon injuries in children [14–23], while concomitant fracture of the neck and shaft of the femur is an even more unusual injury [1–7]. Bennet et al. [7] reported only one case of ipsilateral hip and femoral shaft fracture in children, as compared to 41 such injuries in adults. In 2008, Agarwal et al. [1] stressed the rarity of this concomitant injury, which can be gauged from searching the indexed English literature. They found only seven cases of such injuries in children less than 12 years of age in the literature. They reported a total of nine cases of such injuries, which included two cases of their own. In this work, we have documented three additional reports that we found in the literature but were missed by Agarwal et al. [1]; two were published in the English literature in 1983 and 2006, respectively [9, 10], while the other was published in German in 1986 [11]. These three reports were published before the report of Agarwal et al. (see Table 1).

**Table 1:** Patients demographics

Case	CaseAuthor	Age (years)/ sex	Side	Mode of injury	Femoral neck fx	Shaft fracture	Associated injuries	Management	Union (months)	Coxa vara	Avascular necrosis	Follow-up (months)	Result
1	Present series (case 1)	8/F	Left	Road crash	Cervicotrochanteric (Delbet type III; AO type II)	Sub trochanteric		1 screw & 3 Kirshner wires for the neck fracture, 5-hole plate and 3 screws for the shaft fracture	4	No	No	52	Good Ratliff score. Clinically good with a 10° varus deformity at the subtrochanteric level in the femur
2	Present series (case 2)	8/M	Right	Road crash	Intertrochanteric (Delbet type IV; AO type II)	Distal third	Lateral condyle fracture of the right humerus, distal radius fracture of left wrist fx, subdural hemorrhage of brain, ipsilateral sciatic nerve palsy (peroneal division)	2 screws for the neck fracture, 6-hole plate and screws for the shaft fracture	3	Yes	No	25	Good Ratliff score. Clinically good except for only a slightly short neck and a 1.5 cm leg length discrepancy
3	Akahane	2/F	Right	Road crash	Transepiphyseal (Delbet type Ib; AO epiphyseal type 1)	Distal third		Open reduction and internal fixation with 2 smooth Kirshner wires for neck fracture, hip spica cast. A-frame orthosis for the capital epiphysis	1.5	Yes	Yes (mild)	24	Clinically good, with some coxa vara and premature physeal closure of the medial segent at the 2-year follow-up
4	Cannon	2/F	Left	Fall from height	Transepiphyseal (Delbet type Ia; AO epiphyseal type I)	Midshaft		Closed reduction and hip spica cast for the neck fracture, and 4-hole plate screws for the shaft fracture	2	No	No	12	Excellent, except for some coxa vara
5	Schwarz	10/M	Right		Cervicotrochanteric (Delbet type III; AO type II)	Distal epiphyseal		1 screw and 2 pins for the neck fracture on day 23, conservative for the distal physeal fracture	No comment	Yes	Yes (severe)	188	Aspherical head, coxa vara, short femoral neck, reversed articulotrochantric distance. Valgus and recurvatum deformity of the distal femur

Femoral neck fractures in children are well known for their sinister nature and potential for complications, especially avascular necrosis, which poses the most serious problem and has been reported to have a variable incidence [15, 16, 18–22]. It depends on several factors, such as the degree of initial displacement [15, 17–19, 21, 23], the type of fracture [15, 24, 25], and the timing of surgery [16, 24].

Upon reviewing all nine cases documented by Agarwal et al. [1], six of the femoral neck fractures they reported were cervicotrochanteric (Delbet type III; AO type II), one was transepiphyseal (Delbet type I; AO epiphyseal type 1), another was intertrochanteric (Delbet type IV Delbet; AO type III), while the fracture type was insufficiently described to be able to establish it for the other case. The level of concomitant femoral shaft fracture was midshaft in six cases, distal third in one, and the description was insufficient to determine the level in two cases [1–7].

In this study, we have reported an additional five cases (two from our experience and three more reports fould in the literature). The type of fracture neck femur was transepiphyseal (Delbet type I; AO epiphyseal type I) in two cases, cervicotrochanteric (Delbet type III; AO type II) in two cases, and intertrochanteric (Delbet type IV; AO type III) in one case. If all fourteen cases are considered together, the most common type of femoral neck fracture, when associated with ipsilateral femoral shaft injury, is Delbet type III (AO type II) fracture (eight cases).

Although it is not easy to define the mechanism of trauma, we believe that the mechanism of injury must be high-energy impact at both fracture sites simultaneously. We do not think that sequential application of high-energy forces after a time interval can produce another fracture on top of an already unstable previous fracture.

Various treatment modalities have been tried for these injuries—operative, conservative, or a combination of both. Although good results have been reported with all of these treatment modalities in the literature [1–7], the authors advocate operative stabilization of these injuries to reduce the risk of further displacement of the fracture and allow early mobilization [1, 11]. However, we believe that open reduction and internal fixation of the femoral shaft fracture is the only treatment that permits control over and avoids worsening of the ipsilateral femoral neck fracture; we do not favor it solely because it permits early mobilization. We applied a hip spica cast in both of our cases to promote stability at the fracture site.

We recommend prior fixation of the femoral shaft fracture in these injuries, without attempting to reduce the femoral neck fracture initially, as it provides for more easy subsequent manipulation, reduction, and fixation of the ipsilateral femoral neck fracture. In both of our cases, we fixed the femoral shaft fracture with a plate and screw, and followed this with closed reduction and internal fixation of the femoral neck fracture. However, in one case (case 1), reduction of the subtrochanteric fracture was lost due to proximal screw back-out resulting from inadequate fixation of the proximal fragment, which in turn led to a 10° varus deformity of the proximal femur. In this case, we should have considered the distance between the two fractures to be a prognostic factor, and the possibility of using a low-profile plate that can be modeled in the proximal part to allow introduction of a screw through the proximal hole of the plate into the femoral neck if the two fractures are too close together. We do not need absolutely stable osteosynthesis, because in children younger than ten years it is always advisable to create a spica cast.

At the 2-year follow-up, there was a residual 10° varus deformity of the proximal femur without leg length discrepancy, although leg length discrepancy was noted as being a problem in case 2; this could have occurred after plate and screw fixation when treating the femoral shaft fracture in the child.

Agarwal et al. [1] also noted that a combination of ipsilateral femoral neck and shaft fracture in children had a low incidence of complications. They proposed that the fractures that occur at the two sites in the same limb probably have a symbiotic effect, and that the impact energy was distributed at the time of the initial trauma, thus preventing extreme damage to either of the fracture sites. However, in their report, they only noted cervicotrochanteric and intertrochanteric types of femoral neck fracture, which are less prone to avascular necrosis than the transepiphyseal or transcervical types. In our review of an additional five cases, two had avascular necrosis of the capital epiphysis: one was a transepiphyseal fracture with major displacement (Delbet type Ib) [9] which was treated with open reduction and internal fixation, while the other was a cervicotrochanteric fracture [11] diagnosed on day 9 and operated on day 23 after the initial injury. We do not agree with the view that a symbiotic effect at the time of initial trauma plays a significant role in the prognosis of these injuries, because our study showed relatively high complication rates with transepiphyseal and transcervical femoral neck fractures, which were not presented in the previous literature.

We believe that prior fixation of the femoral shaft fracture in these injuries simplifies the subsequent manipulation, reduction, and fixation of the ipsilateral femoral neck fracture, and that the results of these injuries depend on the severity of the initial trauma, the fracture type, and the timing of surgery.
